# Supplementary material for: Ginsenoside Rk1 bioactivity: a systematic review
Source: PeerJ. 2017 Nov 17;5:e3993. doi: 10.7717/peerj.3993 (PMC5695252; doi:10.7717/peerj.3993)
Supplement: Table S1 [file peerj-05-3993-s002.docx]

**Supplement Table 1.** Search strategy of our systematic review.

| Database | Search strategy |
| --- | --- |
| PubMed | Rk1 AND (ginseng OR panax OR ginsan OR "jen shen" OR shinseng OR "ren shen" OR renshen OR schinseng OR ninjin OR ginsenoside OR gingilone OR panaxoside OR protopanaxa OR protopanaxadiol OR protopanaxatriol OR panaxagin OR ginsenol OR ginsenine) |
| ISI and Scopus | Rk1 AND ginseng Rk1 AND panax Rk1 AND ginsan Rk1 AND jen shen Rk1 AND shinseng Rk1 AND ren shen Rk1 AND renshen Rk1 AND schinseng Rk1 AND ninjin Rk1 AND ginsenoside Rk1 AND gingilone Rk1 AND panaxoside Rk1 AND protopanaxa Rk1 AND protopanaxadiol Rk1 AND protopanaxatriol Rk1 AND panaxagine Rk1 AND ginsenol Rk1 AND ginsenine |
| WHO-GHL, VHL and SIGLE | Rk1 AND (ginseng OR panax OR ginsan OR "jen shen" OR shinseng OR "ren shen" OR renshen OR schinseng OR ninjin OR ginsenoside OR gingilone OR panaxoside OR protopanaxa OR protopanaxadiol OR protopanaxatriol OR panaxagin OR ginsenol OR ginsenine) |
| POPLINE | Rk1 AND (ginseng OR panax OR ginsan OR jen shen OR shinseng OR ren shen OR renshen OR schinseng OR ninjin OR ginsenoside OR gingilone OR panaxoside OR protopanaxa OR protopanaxadiol OR protopanaxatriol OR panaxagin OR ginsenol OR ginsenine) |
| Google Scholar | With all of the words: Rk1 With at least one of the word: ginseng panax ginsan jenshen shinseng renshen schinseng ninjin ginsenoside gingilone panaxoside protopanaxa protopanaxadiol protopanaxatriol panaxagin ginsenol ginsenine |
