# Supplementary material for: Ginsenoside Rk1 bioactivity: a systematic review
Source: PeerJ. 2017 Nov 17;5:e3993. doi: 10.7717/peerj.3993 (PMC5695252; doi:10.7717/peerj.3993)
Supplement: Table S2 [file peerj-05-3993-s003.docx]

| **Supplement Table 2.** Evaluation of the quality of evidence of the included studies. | | | | | | | | | |
| --- | --- | --- | --- | --- | --- | --- | --- | --- | --- |
| **Authors** | **GRADE Factors** | | | | | | | | |
|  | **Study design** | **Study limitation** | **Inconsistency** | **Indirectness** | **Imprecision** | **Publication bias** | **Moderate/Large effect size** | **Dose effect** | **Overall quality** |
| Ko *et al.*, 2009 | *In vitro* | √ | √ | √ | √ | √ | √ | √ | ⊕⊕⊕⊕ |
| Lee *et al*., 2014 | *In vitro* | √ | √ | √ | √ | √ | √ | √ | ⊕⊕⊕⊕ |
| Toh *et al*., 2011 | *In vitro* | √ | √ | √ | √ | √ | √ | √ | ⊕⊕⊕⊕ |
| Kim *et al.*, 2008 | *In vitro* | √ | √ | √ | √ | √ | √ | √ | ⊕⊕⊕⊕ |
| Park *et al.*, 2002 | *In vitro* | √ | √ | √ | √ | √ | √ | Unclear: Not reported | ⊕⊕⊕⊕ |
| Lim *et al.*, 2009 | *In vitro* | √ | √ | √ | √ | √ | √ | Unclear: Not reported | ⊕⊕⊕⊕ |
| Kim *et al.*, 2009 | *In vitro* | √ | √ | √ | √ | √ | √ | √ | ⊕⊕⊕⊕ |
| Kim *et al.*, 2012 | *In vitro* | √ | √ | ×: Indirectness in outcomes | √ | √ | √ | √ | ⊕⊕⊕ |
| Ju *et al.*, 2012 | *In vitro* | √ | ×: Statistical error | √ | √ | √ | √ | √ | ⊕⊕⊕ |
| Liu *et al.*, 2007 | *In vitro* | √ | √ | √ | √ | √ | √ | Unclear: Not reported | ⊕⊕⊕⊕ |
| Lee *et al.*, 2009 | *In vitro* | √ | √ | √ | √ | √ | √ | √ | ⊕⊕⊕⊕ |
| Kim *et al.*, 2010 | *In vivo* | √ | √ | ×: The outcomes considered do not fully answer our questions. | √ | √ | √ | √ | ⊕⊕⊕ |
| Maeng *et al.*, 2013 | *In vitro* and *In vivo* | √ | √ | √ | √ | √ | √ | √ | ⊕⊕⊕⊕ |
| Kang *et al.*, 2007 | *In vitro* | √ | √ | ×: The outcomes considered do not fully answer our questions. | √ | √ | √ | √ | ⊕⊕⊕ |
| Kang *et al.*, 2006 | *In vitro* | √ | √ | √ | √ | √ | √ | × | ⊕⊕⊕⊕ |
| Lee *et al.*, 2010 | *In vitro* | √ | √ | ×: The outcomes considered do not fully answer our questions | √ | √ | √ | × | ⊕⊕⊕ |
| Kim *et al.*, 2013c | *In vitro* | √ | √ | ×: Combined outcome. | √ | √ | √ | √ | ⊕⊕⊕ |
| Bao *et al.*, 2005 | *In vitro* and  *In vivo* | √ | √ | ×: Combined outcome. | √ | √ | √ | √ | ⊕⊕⊕ |
| Park *et al.*, 2015 | *In vitro* and  *In vivo* | √ | √ | ×: Combined outcome. | √ | √ | √ | √ | ⊕⊕⊕ |
| Ponnuraj *et al*., 2014 | *In vitro* | √ | √ | ×: Combined outcome. | √ | √ | √ | √ | ⊕⊕⊕ |
| Siddiqi *et al.*, 2014 | *In vitro* | √ | √ | ×: Combined outcome. | √ | √ | √ | √ | ⊕⊕⊕ |
| Jing *et al.*, 2006 | *In vivo* | √ | √ | ×: Combined outcome. | √ | √ | √ | √ | ⊕⊕⊕ |
| Ahn *et al.*, 2016 | *In vitro* | √ | √ | ×: Combined outcome. | √ | √ | √ | √ | ⊕⊕⊕ |
| Hu *et al*., 2017 | *In vivo* | √ | √ | √ | √ | √ | √ | √ | ⊕⊕⊕⊕ |
| Kwak *et al*., 2016 | *In vitro* | √ | √ | √ | √ | √ | √ | √ | ⊕⊕⊕⊕ |
| Quan *et al*., 2015 | *In vitro* | √ | √ | √ | √ | √ | √ | √ | ⊕⊕⊕⊕ |
| Xue *et al*., 2017 | *In vitro* | √ | √ | √ | √ | √ | √ | √ | ⊕⊕⊕⊕ |
| Ryu *et al*., 2017 | *In vitro* | √ | √ | √ | √ | √ | √ | √ | ⊕⊕⊕⊕ |

Grade Factors: √, No Serious Limitations; X, Serious Limitations (or not present for moderate/large effects size, dose effect size; dose effect); Unclear, Unable to rate item based on available information. For Overall Quality of Evidence: ⊕, very low; ⊕⊕, low; ⊕⊕⊕, moderate; ⊕⊕⊕⊕, high.
